# Improvement in the Biosynthesis of Antioxidant-Active Metabolites in In Vitro Cultures of *Isatis tinctoria* (Brassicaceae) by Biotechnological Methods/Elicitation and Precursor Feeding

**DOI:** 10.3390/antiox12051111

**Published:** 2023-05-17

**Authors:** Natalizia Miceli, Inga Kwiecień, Noemi Nicosia, Jasmine Speranza, Salvatore Ragusa, Emilia Cavò, Federica Davì, Maria Fernanda Taviano, Halina Ekiert

**Affiliations:** 1 Department of Chemical, Biological, Pharmaceutical and Environmental Sciences, University of Messina, Viale Ferdinando Stagno d’Alcontres 31, 98166 Messina, Italy; emilia.cavo@unime.it (E.C.); federica.davi@unime.it (F.D.); mtaviano@unime.it (M.F.T.); 2 Department of Pharmaceutical Botany, Faculty of Pharmacy, Jagiellonian University Medical College, Medyczna 9 Str., 30-688 Krakow, Poland; halina.ekiert@uj.edu.pl; 3 Foundation “Prof. Antonio Imbesi”, University of Messina, Piazza Pugliatti 1, 98122 Messina, Italy; nicosia.noemi@hsr.it (N.N.); js1101@leicester.ac.uk (J.S.); 4 Division of Neuroscience, Vita Salute San Raffaele University, 20132 Milan, Italy; 5 Department of Genetics and Genome Biology, University of Leicester, University Road, Leicester LE1 7RH, UK; 6 PLANTA/Research, Documentation and Training Center, Via Serraglio Vecchio 28, 90123 Palermo, Italy; sragusa@unicz.it

**Keywords:** *Isatis tinctoria*, in vitro cultures, elicitors, precursor feeding, phenolic compounds, antioxidant activity

## Abstract

This study aimed to establish the in vitro shoot culture of *Isatis tinctoria* L. and its ability to produce antioxidant bioactive compounds. The Murashige and Skoog (MS) medium variants, containing different concentrations (0.1–2.0 mg/L) of benzylaminopurine (BAP) and 1-naphthaleneacetic acid (NAA) were tested. Their influence on the growth of biomass, accumulation of phenolic compounds, and antioxidant potential was evaluated. To improve the phenolic content, agitated cultures (MS 1.0/1.0 mg/L BAP/NAA) were treated with different elicitors, including the following: Methyl Jasmonate, CaCl_2_, AgNO_3_, and yeast, as well as with L-Phenylalanine and L-Tyrosine—precursors of phenolic metabolites. The total phenolic content (TPC) of hydroalcoholic extracts (MeOH 70%) obtained from the biomass grown in vitro was determined spectrophotometrically; phenolic acids and flavonoids were quantified by RP-HPLC. Moreover, the antioxidant potential of extracts was examined through the DPPH test, the reducing power, and the Fe^2+^ chelating assays. The biomass extracts obtained after 72 h of supplementation with Tyr (2 g/L), as well as after 120 and 168 h with Tyr (1 g/L), were found to be the richest in TPC (49.37 ± 0.93, 58.65 ± 0.91, and 60.36 ± 4.97 mg GAE/g extract, respectively). Whereas among the elicitors, the highest TPC achieved was with CaCl_2_ (20 and 50 mM 24 h), followed by MeJa (50 and 100 µM, 120 h). The HPLC of the extracts led to the identification of six flavonoids and nine phenolic acids, with vicenin-2, isovitexin, syringic, and caffeic acids being the most abundant compounds. Notably, the amount of all flavonoids and phenolic acids detected in the elicited/precursor feeding biomass was higher than that of the leaves of the parental plant. The best chelating activity was found with the extract of biomass fed with Tyrosine 2 g/L, 72 h (IC_50_ 0.27 ± 0.01 mg/mL), the strongest radical scavenging (DPPH test) for the extract obtained from biomass elicited with CaCl_2_ 50 mM, after 24 h of incubation (25.14 ± 0.35 mg Trolox equivalents (TE)/g extract). In conclusion, the in vitro shoot culture of *I. tinctoria* supplemented with Tyrosine, as well as MeJa and/or CaCl_2_, could represent a biotechnological source of compounds with antioxidant properties.

## 1. Introduction

The indigo dye plant *Isatis tinctoria* L. (woad) is a biennial herbaceous species belonging to the Brassicaceae family and growing spontaneously in mild and sunny climates such as the Middle East and Central Asia and even extending to the Mediterranean [1]. Since ancient times, this plant has been appreciated because of its healing and medicinal properties, and thus it is recommended for the treatment of wounds, ulcers and tumors, hemorrhoids, snakebites, and inflammatory ailments [2]. Nowadays, *I. tinctoria* is one of the top-selling herbal medicines in East Asia; therefore, it is widely employed in traditional Chinese medicine due to its notable anti-influenza efficacy, such as severe acute respiratory syndrome (SARS) and novel swine-influenza A (H1N1) [3]. This plant has also been recognized as a pharmacopoeial plant in Europe over the past decades [4,5].

At this time, an escalating demand for natural compounds has prompted a renewed interest in this plant, leading to the investigation of its phytochemical and biological profile. A remarkable number of studies have displayed a variety of pharmacological properties, including anti-inflammatory, antiviral, antioxidant, and antitumor activity imputable to the synergistic effect of multiple bioactive compounds contained in its extracts [6].

Previously, studies mainly focused on the lipophilic extract from *I tinctoria*, highlighting its strong anti-inflammatory [7] and anti-cancer properties ascribed to alkaloids, tryptanthrin, and indirubin [1]. However, further in vitro and in vivo investigations on polar extracts (MeOH 70%) obtained from the leaves of this species growing wild in Sicily (Italy) demonstrated that the antioxidant activity of *I. tinctoria* is imputable to flavonoids and phenolic acids which are thought to be, together with alkaloids, the primary pharmacological active constituents [8,9].

Although rich in bioactive compounds, the phytochemical profile of *I. tinctoria* is strongly influenced by endogenous and exogenous factors, including geographical location, climatic fluctuations, and soil conditions, which may impair its therapeutic efficacy [10]. From this perspective, plant cell and organ culture via the implementation of biotechnological strategies represent a valuable alternative for the sustainable production of secondary metabolites of pharmaceutical interest with standardized quality. The biotechnology approach provides the opportunity to cultivate different species by regulating conditions such as temperature, light, humidity, and pH regardless of the environment, with the advantage of obtaining higher concentrations of metabolites compared to the traditional method of breeding in the soil [2]. Several biotechnological strategies have been successfully employed to enhance the synthesis and accumulation of high-added value compounds in various plant in vitro cultures, among which elicitation is one of the most promising also due to its low cost, easy reproducibility, and ability to manipulate biochemical and metabolic pathways [11].

To date and to the best of our knowledge, biotechnological studies on *I. tinctoria* have focused on the in vitro production of bioactive secondary metabolites, which are limited to hairy root cultures [2,12,13,14,15,16]; however, no studies have been conducted on in vitro shoot cultures as potential sources of bioactive compounds.

The purpose of the present study was to assess the optimal growth conditions by applying biotechnological strategies to enhance the production and accumulation of antioxidant bioactive compounds in in vitro shoot cultures of *I. tinctoria*. Since the choice of medium composition and plant growth regulators (PGRs) may play a crucial role in biomass growth and metabolite increase, the effect of different concentrations of 6-benzylaminopurine (BAP) and 1-naphthaleneacetic acid (NAA) on the biomass increase, accumulation of phenolic compounds, and antioxidant potential were evaluated. To improve the phenolic content, agitated cultures were treated with different elicitors, including the following: Methyl Jasmonate, CaCl_2_, AgNO_3_, and yeast, as well as with L-Phenylalanine and L-Tyrosine—aromatic amino acids used as biosynthetic precursors of phenolic compounds. The phenolic content of hydroalcoholic extracts (MeOH 70%), obtained from the biomass grown in vitro, was determined spectrophotometrically. The phenolic acids and flavonoids were quantified by RP-HPLC; the antioxidant potential was examined through the 2,2-diphenyl-1-picrylhydrazyl (DPPH) test, the reducing power, and the ferrous ion (Fe^2+^) chelating assays.

## 2. Materials and Methods

### 2.1. Establishment of In Vitro Cultures

Seeds of *I. tinctoria* wildly growing in Acireale (Catania, Sicily, Italy) were picked in May 2018 and applied to establish in vitro shoot cultures. Voucher specimens are deposited in the Herbarium of the Department of Scienze della Salute, University “*Magna Graecia*” of Catanzaro (Italy), under accession number no. 327/11.

Seeds were surface rinsed with distilled water (dH_2_O) and detergent for 20 min and sterilized via 0.1% Mercury chloride II (HgCl_2_) treatment for different ranges of time (5, 10, 15, 20, and 30 min) to define the optimal one. After rising with sterile dH_2_O, *I. tinctoria* seeds were transferred onto Murashige and Skoog (MS) medium [17] previously supplemented with 3% (*w*/*v*) sucrose, 1 mg/L 6-benzylaminopurine (BAP), and 0.5 mg/L 1-naphthaleneacetic acid (NAA) as plant growth regulators and solidified with 0.8% agar (*w*/*v*). After germination, formed shoot cultures were subcultured every two weeks. Two or three leaf rosettes were transferred to each flask. Cultures were maintained in constant white LED light (ca. 16 µmol/m^2^s) at 25 ± 1 °C.

### 2.2. Optimization of Growth Conditions of Different Types of Cultures

#### 2.2.1. Stationary Culture

Different PGRs were tested to define the optimal medium composition for growth conditions and cultivation of *I. tinctoria* shoot cultures [18]. The following PGRs, alone or combined, were applied to MS agar medium: 6-benzylaminopurine (BAP), kinetin (KIN), 1-naphthaleneacetic acid (NAA), and indole-3-butyric acid (IBA).

Having established BAP and NAA as the best PGRs, further preliminary studies were carried out to identify the optimal concentration. Thus, various combinations of BAP and NAA were applied to the agar medium (five replicates each), including the following: 2 mg/L BAP + 1 mg/L NAA, 1 mg/L BAP + 1 mg/L NAA, 1 mg/L BAP + 0.5 mg/L NAA, 1 mg/L BAP, 0.5 mg/L BAP, and 1 mg/L NAA. After the experiment, the fresh and freeze-dried weights of biomass were recorded, and the results were expressed as the mean of values ± SD (*n* = 5).

#### 2.2.2. Agitated Culture

Agitated cultures were initiated from 3-week-old in vitro stationary cultures of *I. tinctoria*. The fresh biomass (~0.5 g) was inoculated into 100 mL Erlenmeyer flasks containing MS liquid medium supplemented with the variants of PGRs previously applied in the stationary cultures: 2 mg/L BAP + 1 mg/L NAA, 1 mg/L BAP + 0.5 mg/L NAA, 1 mg/L BAP + 1 mg/L NAA, 1 mg/L NAA, 1 mg/L BAP, and 0.5 mg/L BAP. Six samples of *I. tinctoria* cultures were set for each combination. Cultures were maintained under constant white LED light (ca. 16 µmol/m^2^s) on a rotary shaker (Altel, Cracow, Poland) at 140 rpm at 25 ± 1 °C for two weeks. After the experiment, the fresh and freeze-dried weights of biomass were recorded, and the results were expressed as the mean of values ± SD (*n* = 6).

### 2.3. Elicitation of Cultures

For the elicitation experiment, agitated cultures were chosen. Approximately 1 g of fresh biomass was inoculated into a 250 mL Erlenmeyer flask containing 100 mL of MS liquid medium variant (1.0 mg/L BAP and 1. 0 mg/L NAA). Cultures were maintained on a rotary shaker for 3 weeks as described previously.

#### 2.3.1. Elicitors Preparation

Methyl Jasmonate (MeJa), yeast (YE) extract, Calcium chloride (CaCl_2_), and Silver nitrate (AgNO_3_) were selected as hormonal, biotic, and abiotic elicitors, respectively. MeJa (Sigma-Aldrich, Saint Louis, MO, USA) was dissolved in 95% ethanol, diluted in distilled water (dH_2_O), and filter-sterilized using a 0.22 μm syringe filter (Millex^®^GP; Merck Millipore, Burlington, MA, USA). A stock solution of yeast (YE) extract was prepared according to the Ge and Wu method [19]. A total of 50 g of yeast extract (Duchefa, Biochemie, Haarlem, The Netherlands) was dissolved in distilled water (20 g/100 mL), then mixed with 400 mL of ethanol and allowed to settle for 4 days at 4 °C in a refrigerator. The gummy precipitate was filtered by using a 0.22 μm syringe filter, dissolved in 250 mL of dH_2_O, and autoclaved at 121 °C for 20 min. To determine the total carbohydrate content in the YE extract, a quantitative colorimetric method was performed by using a phenol-sulfuric acid reaction—useful to detect small quantities of sugars and related substances [20,21]. CaCl_2_ and AgNO_3_ solutions were obtained via solubilization in dH_2_O and filter-sterilized using a 0.22 μm syringe filter [22].

#### 2.3.2. Elicitation Procedure

Stock solutions of elicitors were administered to the suspension culture medium on the 22nd day of the growth period. Three different concentrations were used for each elicitor: MeJa was applied to the medium in the final concentrations of 10, 50, 100 μM [23,24]; CaCl_2_ was added to the medium to yield in the final concentrations of 20, 50, 100 mM [21]; AgNO_3_ was added to the medium to yield in the final concentrations of 0.5, 1, 2 mM [25]; YE extract was used in the concentrations of 50, 200, 300 mg/L [23,26]. Shoot cultures grown without elicitors were established as controls (C) and treated with the corresponding amount of dH_2_O or EtOH used to dissolve elicitors. Three series of cultures were set up for both experimental and control samples. Controls and elicited cultures were collected on the 1st (24 h), 2nd (48 h), 5th, (120 h), and 7th (168 h) days since the moment that the elicitor was added.

### 2.4. Precursors Feeding of Cultures

For this experiment, the agitated cultures were used and prepared in the same manner as the elicitor treatment.

L-Tyrosine (Tyr) (Sigma-Aldrich^®^, St Louis, MO, USA) and L-Phenylalanine (Phe) (Merck, Darmstadt, Germany) were selected for this experiment as precursors of phenolic compounds [27,28]. Phe and Tyr were dissolved in dH_2_O and filter-sterilized. For each flask, 10 mL of stock solution was added to the MS medium on the 22nd day of growth to yield the final concentrations of 1 g/L and 2 g/L [28,29]. One set of shoot cultures was maintained as control (C) and filled up with the corresponding amount of dH_2_O used to dissolve precursors. The shoot cultures were harvested in triplicate after the 3rd (72 h), 5th, (120 h), and 7th (168 h) days of cultivation after precursor addition [28,30].

### 2.5. Extracts Preparation

After each experiment, the fresh biomass collected was immediately lyophilized (freeze dryer Labconco Corporation, Kansas City, MO, USA), sequentially extracted in the manner of Taviano et al. [31], and subjected to phytochemical analysis and an antioxidant test. The dried plant material was weighed, pulverized, and treated with dichloromethane (DCM) and 70% methanol (120 mL, three times for 15 min at 50 °C). The hydroalcoholic extracts (MeOH 70%) were pooled and evaporated to dryness in vacuo. The yields of the extracts, accounting for 100 g of dried plant material, are reported in Tables S1–S3.

### 2.6. Phytochemical Analysis

#### 2.6.1. Determination of Total Phenolic Content

The total phenolic content (TPC) of the hydroalcoholic extracts obtained from *I. tinctoria* shoot cultures was determined using the Folin–Ciocalteu method, which considers the calibration curve of gallic acid—the phenol compound used as a standard [32]. Each sample solution (100 μL) was mixed with 0.2 mL Folin–Ciocalteu reagent, 2 mL of distilled water, and 1 mL of 15% Na_2_CO_3_, and the absorbance was measured at 765 nm after 2 h incubation at r.t. using a model spectrophotometer (Shimadzu, Milan, Italy). The TPC was estimated as gallic acid equivalents (GAE) and expressed in mg GAE/g extract Dried Weight (DW) ± SD.

#### 2.6.2. HPLC Analysis

The hydroalcoholic extracts were dissolved in HPLC-grade methanol and then analyzed using a modified HPLC method [33]. An HPLC system (Merck–Hitachi) and a Purospher RP-18e analytical column (4 × 250 mm, 5 μm; Merck) were used. The mobile phase consisted of methanol (A) and 0.5% acetic acid (B) (gradient elution). The flow rate was 1 mL/min. Compounds were estimated using a DAD detector. Qualification and quantification analyses were based on comparisons with the following reference substances: 3,4-dihydroxyphenylacetic acid, 3-hydroxyphenylacetic acid, caftaric acid, caffeic acid, chlorogenic acid, cryptochlorogenic acid, 2-coumaric, 3-coumaric, 4-coumaric acids, dihydrocaffeic acid, ellagic acid, ferulic acid, 4-O-feruloylquinic acid, gallic acid, gentisic acid, hydrocaffeic acid, 4-hydroxybenzoic acid, isochlorogenic acid, isoferulic acid, neochlorogenic acid, protocatechuic acid, rosmarinic acid, salicylic acid, sinapic acid, syringic acid, and vanillic acid (Sigma-Aldrich^®^, St Louis, MO, USA); benzoic, phenylacetic, and cinnamic acids (Sigma-Aldrich^®^, St Louis, MO, USA); apigenin, chrysin, cynaroside, isorhamnetin, hyperoside, luteolin, narigenin, myricetin, populnin, quercetin, quercitrin, rhamnetin, robinin, rutoside, scutellarein, vitexin, wogonoside (Sigma-Aldrich^®^, St Louis, MO, USA); apigenin 5-glucoside, apigenin 7-glucuronide, apigenin 4′-rhamnoside, astragalin, avicularin, baicalin, baicalein, diosmetin, isoquercetin, kaempferol, kaempferol 3-glucoside, kaempferol 3-rhamnoside, kaempferol 7-rhamnoside, kaempferol 4-glucoside, miquelianin, narirutin, quercetin 3-glucuronide, scutellarin, wogonin; (ChromaDex, Irvine, CA, USA); apigetrin, isovitexin, gujaverin, oroxylin A, sculcapflavone II, trifolin, vicenin II (Chemfaces, Wuhan, China).

### 2.7. Antioxidant Activity

#### 2.7.1. Free Radical Scavenging Activity

The free radical scavenging activity of hydroalcoholic extracts obtained from *I. tinctoria* shoot cultures was determined using the DPPH (2,2-diphenyl-1-picrylhydrazyl) method [34]. The extracts were tested at different concentrations (0.0625–2 mg/mL). An aliquot (0.5 mL) of each sample solution was added to 3 mL of daily prepared methanol DPPH solution (0.1 mM). The optical density change at 517 nm was measured 20 min after the initial mixing using a model UV-1601 spectrophotometer (Shimadzu). The scavenging activity was measured as the decrease in absorbance of the sample’s vs. the DPPH control solution. Trolox (0.03–0.1 mg/mL) was used as a standard. The assays were carried out in triplicate, and the results are reported as mg of Trolox equivalents (TE)/g extract ± SD.

#### 2.7.2. Reducing Power

The reducing power of hydroalcoholic extracts obtained from *I. tinctoria* shoot cultures was determined according to the method of Oyaizu [35]. Different amounts of each extract (0.0625–2 mg/mL) in 1 mL solvent were mixed with 2.5 mL of phosphate buffer (0.2 M, pH 6.6) and 2.5 mL of 1% K_3_Fe (CN)_6_. The mixture was incubated at 50 °C for 20 min. The resulting solution was cooled rapidly, mixed with 2.5 mL of 10% trichloroacetic acid, and centrifuged at 1570× *g* for 10 min. The resulting supernatant (2.5 mL) was mixed with 2.5 mL of distilled water and 0.5 mL of 0.1% fresh ferric chloride (FeCl_3_), and the absorbance was measured at 700 nm after 10 min. As a blank, an equal volume (1 mL) of water was mixed with a solution prepared as described above. Ascorbic acid was used as reference. The results were obtained from the average of three independent experiments and are expressed as Ascorbic acid Equivalent (ASE/mL) ± SD.

#### 2.7.3. Ferrous Ion (Fe^2+^) Chelating Activity

The Fe^2+^ chelating activity of hydroalcoholic extracts obtained from *I. tinctoria* in vitro shoot cultures was estimated by the method of Decker and Welch [36]. Briefly, different concentrations of each extract (0.0625–2 mg/mL) in 1 mL solvent were mixed with 0.5 mL of methanol and 0.05 mL of 2 mM FeCl_2_. The reaction was initiated by the addition of 0.1 mL of 5 mM ferrozine. Then, the mixture was shaken vigorously and left standing at room temperature for 10 min. The absorbance of the solution was measured spectrophotometrically at 562 nm. The control contained FeCl_2_ and ferrozine—complex formation molecules. The assays were carried out in triplicate, and the results are expressed as the mean 50% inhibitory concentration (IC_50_) ± SD.

### 2.8. Statistical Analysis

A statistical comparison of the data was carried out by using one-way and two-way analysis of variance (ANOVA) followed by Tukey–Kramer multiple comparisons test via GraphPAD Prism Soft-ware for Science or Statistica 13.3 (TIBCO Software Co., Palo Alto, CA, USA). *p*-values lower than 0.01 were considered statistically significant.

## 3. Results

### 3.1. Establishment of In Vitro Cultures

**Figure 1:**
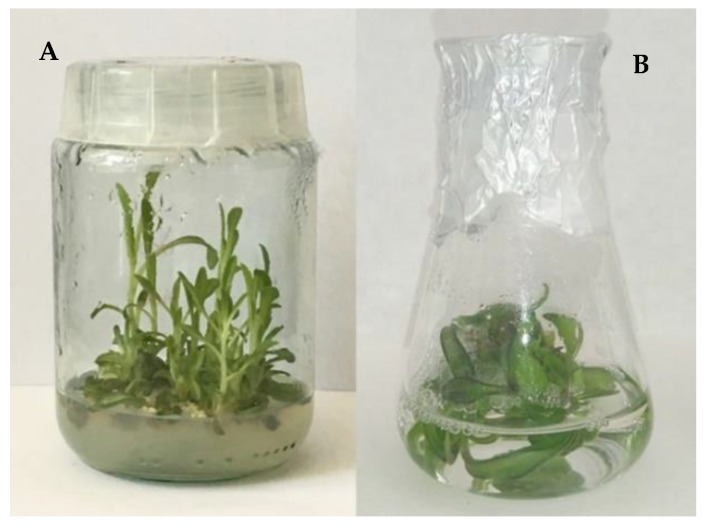
*Isatis tinctoria* in vitro cultures: stationary shoot culture (MS variant BAP/NAA 1.0/1.0 mg/L) (**A**), agitated shoot culture (MS variant BAP/NAA 1.0/0 mg/L) (**B**).

The establishment of *I. tinctoria* in vitro culture using seeds germinated in vitro as explants was successfully reached after 1 month. It was noted that 5 min of 0.1% HgCl_2_ treatment of the seeds is enough to reach a good sterilization standard. The results obtained from this experiment showed that KIN and IBA did not affect the in vitro culture growth positively. On the other hand, the shoot formation was improved after the addition of BAP and NAA. These PGRs were selected to perform subsequent experiments with the aim of optimizing growth conditions (Figure 1A).

### 3.2. Optimization of Growth Conditions of Different Types of Cultures

#### 3.2.1. Agar Culture

The effect of different concentrations of BAP and NAA on the shoot cultures growth were reported in Table 1. The fresh biomass increments of *I. tinctoria* shoot cultures ranged from 1.840 to 3.579-fold. The MS medium variant supplemented with 1 mg/L BAP + 1 mg/L NAA achieved the best culture growth. On the other hand, the one supplemented with 1 mg/L NAA achieved the worst growth. Moreover, good results were obtained on the MS medium variant containing 0.5 mg/L BAP and 1 mg/L BAP + 0.5 mg/L NAA.

#### 3.2.2. Agitated Culture

The fresh biomass increments of *I. tinctoria* agitated cultures (Figure 1B) ranged from 1.964 to 11.663-fold. In Table 2, it is possible to see that the agitated in vitro cultures achieved maximal growth on the MS medium supplemented with 2 mg/L BAP + 1 mg/L NAA. A good biomass increase (10/11-fold) was recorded in the MS medium variant containing 1 mg/L BAP + 1 mg/L NAA and 1 mg/L BAP. Evidently, as in the previous experiment, the variant of MS medium characterized by 1 mg/L NAA was determined to be the worst culture growth.

### 3.3. Elicitation of Cultures

In the case of agitated cultures maintained in a liquid medium, dry biomass is a better parameter to describe the growth of biomass because of the differences in the hydration of fresh biomass tissue. In vitro cultures of *I. tinctoria* elicited by MeJa show increases in dry biomass ranging from 4.2 to 19.5 times. This parameter was higher than the control cultures for all MeJa concentrations used, except 50 and 100 µM on day 5. The time of exposure to this elicitor had a negative effect on biomass growth. In the case of inorganic elicitors, a positive effect was observed in biomass growth, especially at 24 h after adding CaCl_2_ and AgNO_3_ to the medium. Cultures elicited by CaCl_2_ showed very diverse increases in dry biomass, from 2.9- to 20.2-fold. In the case of AgNO_3_, the biomass increases ranged from 5.4 to 18.6 times. After the addition of the YE extract, no visible effect of the incubation time on biomass growth was documented. Cultures showed 4.3- to 7.9-fold increases in dry biomass. The results were comparable to or lower than the values obtained for the control cultures (Table 3). There was no statistically significant interaction between factors (time and concentrations used) for any of the elicitors at α = 0.01. For MeJa and calcium chloride, time was a significant factor (*p* = 0.0021 and *p* = 0.0001, respectively). When silver nitrate was used, its concentration turned out to be a statistically significant factor (*p* = 0.0051).

**Figure 2:**
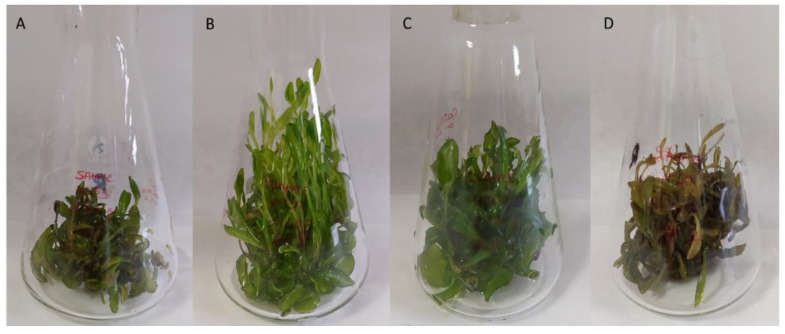
*Isatis tinctoria* in vitro cultures (MS variant BAP/NAA 1.0/1.0 mg/L) after 168 h elicitation by CaCl_2_ 20 mM (**B**), 50 mM (**C**), 100 mM (**D**), and control culture (**A**).

Of the elicitors used, only MeJa in the concentrations applied did not cause any changes in the appearance of the in vitro cultures. Both AgNO_3_ and YE extract resulted in the darkening of the elicited biomass. On the other hand, CaCl_2_ positively affected the appearance and foliage of cultures, causing intensive growth (Figure 2).

### 3.4. Precursors Feeding of Cultures

Cultures whose medium had been administered Phe at a concentration of 1 g/L showed 14.0- to 16.5-fold increases in dry biomass and 13.6- to 18.5-fold increases at a concentration of 2 g/L. No negative effects of incubation time on in vitro culture growth intensity were observed. In comparison, cultures supplemented with Tyr at a concentration of 1 g/L showed 9.6- to 21.7-fold increases and 4.5- to 15.3-fold dry biomass increases at a concentration of 2 g/L. In the case of Tyrosine supplementation, some negative effects on growth were observed when compared to control cultures. This is particularly evident after 168 h of incubation with 1 g/L Tyr and 120 and 168 h of incubation with 2 g/L Tyr (Table 4). No significant differences were found between the mean values at α = 0.01.

**Figure 3:**
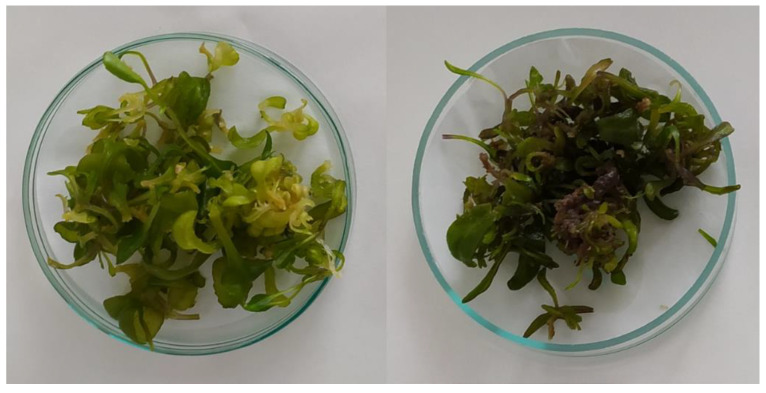
*Isatis tinctoria* in vitro cultures (MS variant BAP/NAA 1.0/1.0 mg/L) 120 h after the addition of Phenylalanine (**left**) and Tyrosine (**right**) at a concentration of 1 g/L.

Administration of Tyr to the medium as a precursor, in contrast to Phe, caused the darkening of the biomass (Figure 3). This effect was observed on day 5 (120 h) and day 7 (168 h) after administering tyrosine for both concentrations.

### 3.5. Phytochemical Investigations

#### 3.5.1. Determinations of Total Phenolic Content

The total phenolic content (TPC) of hydroalcoholic extracts obtained from *I. tinctoria* agitated cultures ranged from 9.604 ± 0.555 mg GAE/g in the extract prepared from in vitro culture grown on MS variant with BAP/NAA 0/1.0 mg/L to 14.451 ± 1.043 mg GAE/g in the extract obtained from *I. tinctoria* biomass grown on MS medium variant with BAP/NAA 1.0/1.0 mg/L (Table 5).

The latter variant, due to it achieving the best results, was selected for further experiments aimed at increasing phenolic compounds in in vitro grown biomass.

Elicitation and precursor feeding were utilized as strategies to improve the in vitro production of secondary metabolites.

Table 6 shows the TPC of extracts obtained from in vitro shoot cultures of *I. tinctoria*, grown in a medium supplemented with different doses and subjected to different incubation time of MeJa, abiotic elicitor (CaCl_2_, AgNO_3_), biotic elicitor (yeast extract), and amino acid precursors of phenolic compounds (L-Phenylalanine and L-Tyrosine).

As shown in Table 6, the MeJa treatment, for 24 or 48 h, with the doses 50 and 100 µM caused a significant (*p* < 0.0001) increase (ranging from 1.29 to 1.39-fold) in TPC in the extracts obtained from elicited biomass compared to cultures not subject to elicitation (control). A dose of 100 µM, after 48 h of exposure, determined the best increase in TPC (1.39-fold) with respect to the control. On the contrary, a decrease in TPC, in comparison to the control, was highlighted in extracts of biomass supplemented for 120 and 168 h with MeJa. In any case, among the extracts of biomass elicited with MeJa, the richest in TPC was the one from biomass supplemented for 120 h with a dose of 50 µM (33.36 ± 1.65 mg GAE/g extract) (Table 6).

The treatment for 24 h (at the doses of 20 and 50 mM) or 48 h (50 mM) with the abiotic elicitor CaCl_2_ determined a significant (*p* < 0.0001) enhancement in TPC ranging from 1.20 to 1.3-fold compared to the control biomass extract. The most effective dose was that of 50 mM, which, after 48 h of incubation, led to a significant (*p* < 0.0001) 1.3-fold increase in comparison to the control (26.51 ± 0.54 mg GAE/g extract and 20.23 ± 0.86 mg GAE/g extract, respectively). Among the extracts of shoot culture elicited with CaCl_2_, the richest in TPC were the extracts of biomass, which were supplemented for 24 h, with the doses of 20 and 50 mM (30.43 ± 1.06 and 29.87 ± 1.07 mg GAE/g extract). As observed in the elicitation with MeJa, a decrease in TPC, in comparison to the control, after supplementation for 120 and 168 h with CaCl_2_ was highlighted (Table 6). Finally, the treatment with the abiotic elicitor (AgNO_3_) and with the biotic elicitor (YE extract) determined, in the range of tested concentrations, a decrease in TPC in comparison to that of the control biomass extracts (Table 6).

The use of precursors, such as the amino acids Phenylalanine and Tyrosine, determined an increase in TPC in all extracts at each concentration and incubation time. In particular, treatment for 72 h with both doses of 1 and 2 g/L of Phe, as well as for 168 h with the highest concentration (2 g/L), resulted in a significant increase in TPC in the extracts of biomass fed with this precursor compared to the control (*p* < 0.0001); a dose of 2 g/L after 72 h of exposure determined the best increase in TPC (2.04-fold) with respect to the control. Among the extracts of shoot culture supplemented with Phe, the richest in TPC was the one from biomass treated for 168 h with the highest dose (41.43 ± 6.45 mg GAE/g extract). No significant enhancement in TPC was highlighted neither after 120 h of supplementation with both doses of Phe nor after 168 h with 1 g/L (Table 7).

For the extracts obtained from biomass grown on media supplemented with Tyrosine, a significant (*p* < 0.0001) increase (ranging from 1.23 to 2.78-fold) with respect to the control biomass extract was observed for all doses and incubation times, except for the 2 g/L dose after 168 h of exposition. In comparison to the control, the highest increase in TPC (2.78-fold) was facilitated by a dose of 2 g/L after 72 h of exposure. Additionally, the biomass extracts richest in TPC were those supplemented with 1 g/L of Tyr for 120 and 168 h (58.65 ± 0.91 and 60.36 ± 4.97 mg GAE/g extract), followed by the biomass extract supplemented with the highest dose of Tyr for 120 h (49.37 ± 0.93 mg GAE/g extract) (Table 7).

#### 3.5.2. HPLC Analysis

The extracts from the biomass of the cultures elicited were found to contain protocatechuic, chlorogenic, neochlorogenic, vanillic, caffeic, syringic, p-coumaric, ferulic, and sinapic acid of phenolic acids, and vicenin-2, isovitexin, apigetrin, apigenin, quercetin, quercitrin belonging to flavonoids.

**Figure 4:**
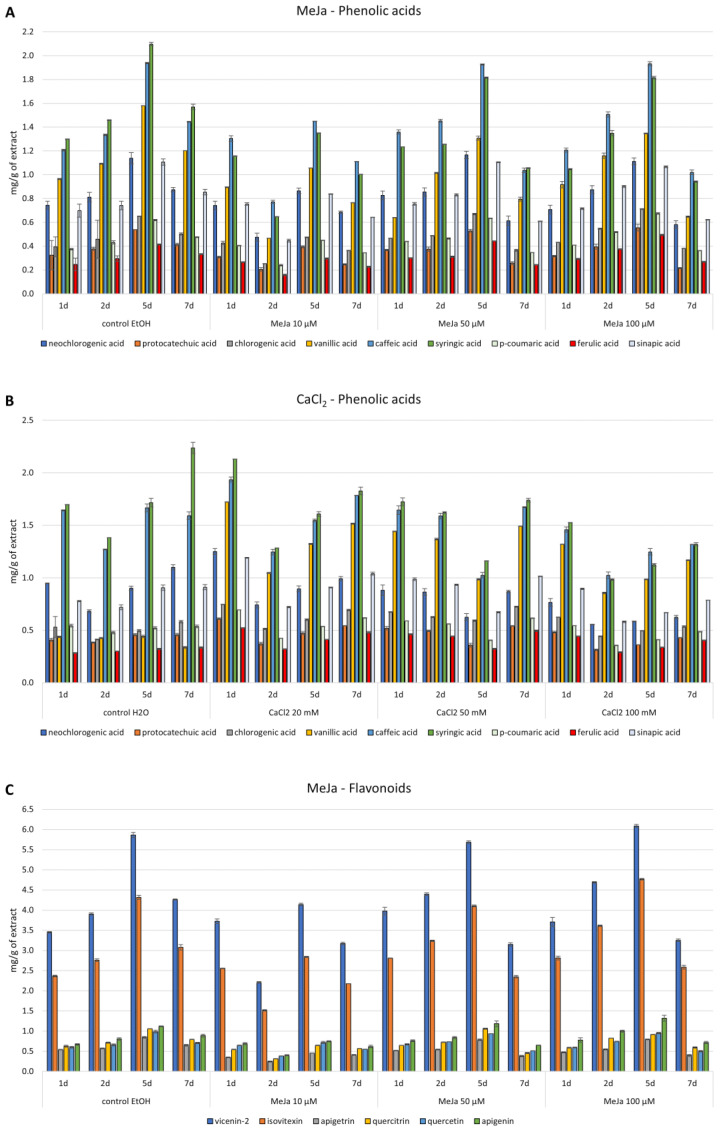

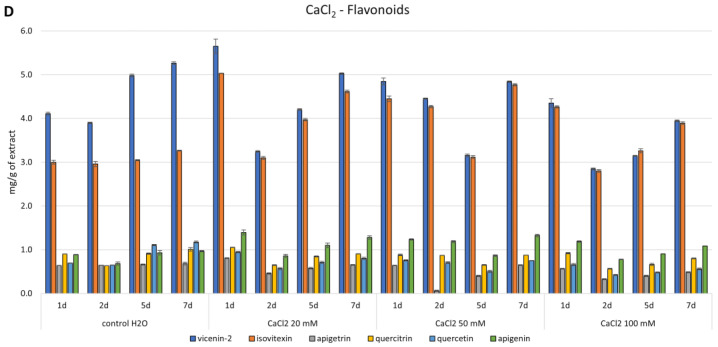
Content of phenolic acids and flavonoids, determined by RP-HPLC analysis, in *Isatis tinctoria* in vitro culture extracts elicited by Methyl Jasmonate—MeJa (**A**,**C**) and Calcium chloride—CaCl_2_ (**B**,**D**). Values are expressed as the mean ± SD (*n* = 3).

Administering MeJa at the lowest concentration used (10 µM) resulted in a decrease in the content of the detected phenolic acids in the biomass in practically all samples. Higher concentrations, 50 and 100 µM, resulted in some increases in the content of neochlorogenic, chlorogenic, p-coumaric, ferulic, and sinapic acids during the majority of the time points studied (1.02 to 1.27-fold) (Figure 4A).

The supplementation of CaCl_2_ in the medium resulted in an increase in the content of chlorogenic and ferulic acid throughout the experiment. The largest increases were 1.84 times for ferulic acid and 1.52 times for chlorogenic acid, respectively. However, the highest confirmed increases in content were for vanillic acid—2.46–4.50 times at a concentration of 20 mM and 2.21–4.43 times at a concentration of 50 mM. At lower concentrations of CaCl_2_, a slight increase in the content of protocatechuic, p-coumaric, and sinapic acids was also confirmed (Figure 4B).

The addition of AgNO_3_ to the cultures resulted in a decrease in the detected phenolic acid content. Among the 9 acids, only the content of vanillic and ferulic acids increased after the use of this elicitor (1.72–2.33 and 1.01 to 1.28 times, respectively). In the case of chlorogenic acid, on the second day after administration of AgNO_3_ at concentrations of 0.5 and 1 mM, a slight increase in the content (7 and 11%) was confirmed (Figure S1). Similar results were obtained after using yeast extract as an elicitor. The vanillic acid content more than doubled in all cultures tested. The highest content of this acid (2.50–3.25 times) was confirmed after 7 days of incubation with all concentrations used. The ferulic acid content also increased from 1.07 to 1.25 times. However, among the remaining acids, only slight increases in the chlorogenic acid content were confirmed on day two at the YE concentration of 100 and 300 mg/L and protocatechuic acid at the YE concentration of at100 mg/L (Figure S1).

The results for flavonoids vary widely. Briefly, administering MeJa resulted in an increase in vicenin-2, isovitexin, quercetin, and apigenin on the first and second days after adding the elicitor at a concentration of 50 uM, and an increase in vicenin-2, isovitexin, and apigenin on the first, second, and fifth days after adding the elicitor at a concentration of 100 µM (Figure 4C).

The supplementation of cultures with CaCl_2_ increased the isovitexin content 1.05 to 1.68-fold at 20 mM, 1.02 to 1.48-fold at 50 mM, and 1.07 to 1.42-fold at 100 mM, respectively. For apigenin, these increases were similar and ranged from 1.18 to 1.57 times at 20 mM, 1.37 to 1.74 times at 50 mM, and 1.12 to 1.36 times at 100 mM, respectively. In the case of other flavonoids, an increase in content (in the range of 1.17–1.68 times) was observed only at the lowest elicitor concentration 1 day after its administration (Figure 4D).

The elicitation of cultures with AgNO_3_ increased the apigenin biomass content by up to 38% on the second day at a concentration of 0.5 mM. Additionally, isovitexin was up to 20% at lower elicitor concentrations (0.5 and 1 mM) (Figure S1). Similarly, in the case of elicitation with yeast extract, the content of apigenin and isovitexin increased by 1.02 to 1.60 and 1.05 to 1.29 times, respectively. Furthermore, when using the lowest elicitor concentration (50 mg/L), a 10 to 29% increase in quercitrin content was observed (Figure S1).

The biomass extracts of cultures supplemented with phenylalanine and tyrosine in both concentrations were found to contain the same metabolites as *I. tinctoria* cultures treated with elicitors.

**Figure 5:**
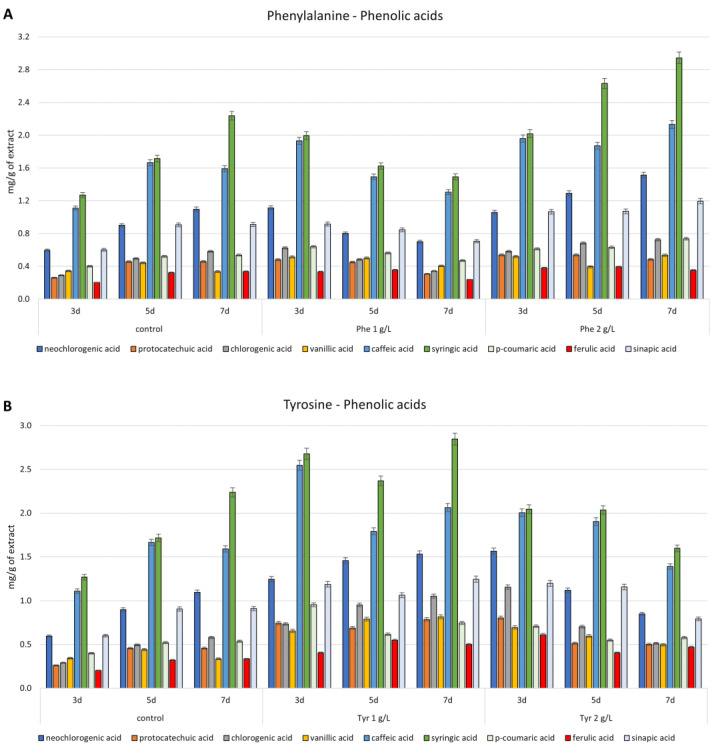

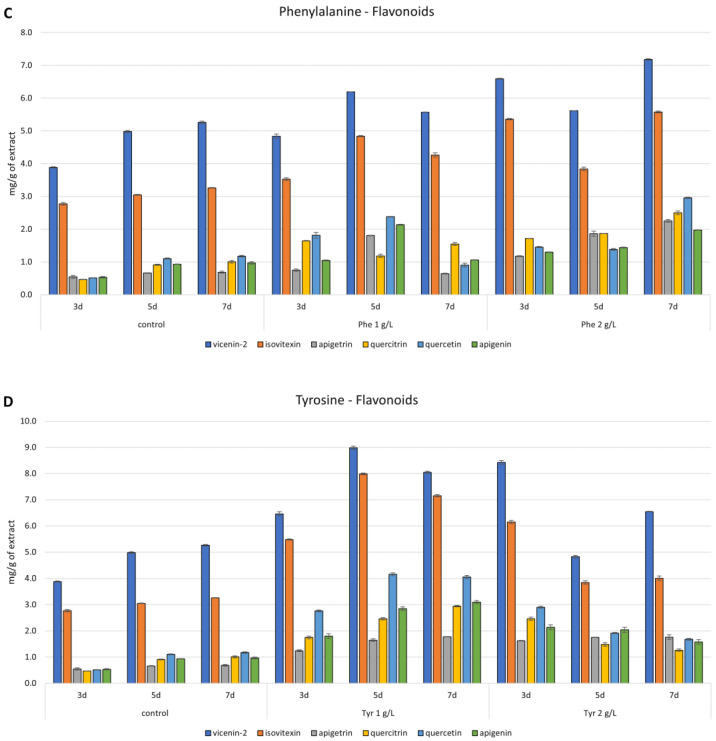
Content of phenolic acids and flavonoids, determined by RP-HPLC analysis, in *Isatis tinctoria* in vitro culture extracts supplemented with Phenylalanine-Phe (**A**,**C**) and Tyrosine—Tyr (**B**,**D**). Values are expressed as the mean ± SD (*n* = 3).

As a result of phenylalanine supplementation at a lower concentration (1 g/L), a 1.50–2.15 times increase in the content of phenolic acids was obtained only on the third day, compared to the control cultures. A slight increase (1.1–1.2 times) in the content of p-coumaric was also confirmed on the fifth day, and for ferulic acid and vanillic acid, on the fifth and seventh day, respectively. With a concentration of 2 g/L of phenylalanine during all time points, an increase in all metabolites was observed. The highest increases obtained (1.52–2.07-fold) were observed on the third day after the administration of the precursor at 1 g/L (Figure 5A).

The effect of phenylalanine administration on the content of flavonoids was more visible. Under the influence of a concentration of 1 g/L, the content of the compounds increased (1.06 to 3.54 times), but only for apigetrine, while the content of quercetin on the seventh day was lower than in the control. The highest increase in quercetin and quercitrin was confirmed on day three (3.54 and 3.53 times, respectively). An increase of more than twofold was also obtained for apigetrine, quercetin, and apigenin on day five after the administration of the precursor. The higher concentration of phenylalanine resulted in respective increases in the content of all detected flavonoids as follows: 1.70 to 3.68 on the third day, 1.13 to 2.81 on the fifth day, and 1.36 to 3.29 on the seventh day. The highest increases were confirmed for apigetrin and quercitrin. More than twofold increases for quercetin and apigenin were also obtained on days three and seven (Figure 5C).

Supplementation with the second aromatic amino acid, tyrosine, gave better results than phenylalanine. After applying a concentration of 1 g/L, the content of individual phenolic acids increased by 1.91 to 2.86 times on the third day, 1.08 to 1.93 on the fifth day, and 1.27 to 2.43 on the seventh day. However, at a concentration of 2 g/L, the contents increased from 1.61 to 3.98 on the third day, from 1.05 to 1.42 on the fifth day, and 0.77 to 1.39 on the seventh day, respectively, where the values for neochlorogenic, chlorogenic, caffeic, syringic, and sinapic acids were below the control values. The highest increases were confirmed for chlorogenic, protocatechuic, and ferulic acids. In the case of tyrosine, as in the case of phenylalanine, with both at a concentration of 1 and 2 g/L, the highest increases in the content of each phenolic acid were obtained on the third day after the administration of the precursors (Figure 5B). Tyrosine feeding has been shown to be the best strategy for flavonoids. Supplementation of this precursor at a concentration of 1 g/L resulted in the following increases in these metabolites at all time points: 1.66 to 5.39 on day three, 1.80 to 3.76 on day five, and 1.53 to 3.45 on day seven, respectively. The highest confirmed increase in content was for quercetin (3.44–5.39 times), quercitrin, and apigenin (over or nearly three times). A concentration of 2 g/L also increased the content of individual flavonoids, which was most pronounced on the third day after the administration of the precursor (from 2.16 to 5.67 times). More than five-fold increases for quercetin and quercitrin were confirmed, while the content of apigenin and apigetrin increased by 4.01 and 2.97 times, respectively. On days five and seven, more than 2.5-fold increases in apigetrine were also obtained (Figure 5D).

### 3.6. Antioxidant Activity

The antioxidant activity was evaluated by using different in vitro assays. The primary antioxidant properties were examined using the DPPH assay, based on the hydrogen atom transfer (HAT) and electron transfer (ET) mechanisms, and the reducing power—an ET-based assay. The secondary antioxidant ability was determined by measuring the ferrous ion (Fe^2+^)-chelating activity.

All the extracts did not manifest any reducing power ability. Table 8 shows the radical scavenging activity (expressed as mg Trolox equivalents (TE)/g extract) and chelating ability (expressed as IC_50_) of the hydroalcoholic (MeOH 70%) extracts obtained from the biomass grown on different MS medium variants. In the DPPH test, the best efficacy was found for the extract from biomass cultured in medium supplemented with BAP/NAA 1.0/0 mg/L and BAP/NAA 1.0/1.0 mg/L (9.808 ± 0.687 mg TE/g extract and 9.653 ± 0.261 mg TE/g extract, respectively). The latter also exhibited the best activity in the ferrous ion (Fe^2+^)-chelating assay, as confirmed by an IC_50_ value equal to 0.640 ± 0.013 mg/mL.

In Table 9, the radical scavenging activity of extracts obtained from biomass elicited with MeJa, CaCl_2_ AgNO_3_, and YE extract is reported. The extracts obtained from biomass elicited with MeJa and CaCl_2_ displayed good radical scavenging properties. The extracts showing the best activity in the DPPH test were those obtained from biomass elicited for 120 h with 50 and 100 µM of MeJa (24.20 ± 0.66 and 24.79 ± 0.38 mg TE/g extract) and those of biomass elicited for 24 h with 50 mM of CaCl_2_ (25.14 ± 0.35 mg TE/g extract). On the other hand, the extracts of biomass treated with AgNO3 showed weak radical scavenging activity (ranging from 11.88 ± 0.19 to 4.25 ± 0.58 mg TE/g extract), as their values were always lower than that of the control. (Table 9).

The extracts of biomass treated with the biotic elicitor (YE extract) for 120 and 168 h displayed moderate activity (from 22.03 ± 0.79 to 12.28 ± 0.01 mgTE/g extract), which was, in any case, lower than that of the extracts obtained from control cultures (Table 9).

The extracts of biomass treated with both precursors (Phenylalanine and Tyrosine) showed moderate activity in the DPPH test (Table 10). Regarding Phe, radical scavenging activity was observed in the extract obtained from shoot culture feeding for 72 h with a dose of 2 g/L of this precursor (19.51 ± 0.55 mg TE/gextract). At the same time, the extracts of biomass treated with 1 and 2 g/L of Phe for 120 h, as well as with 2 g/L for 168 h, showed good activity (19.51 ± 0.55, 21.85 ± 0.15 and 17.44 ± 0.03 mg TE/g extract, respectively), which remains lower than that of the control extracts.

The same trend was observed for the treatment with Tyr. A moderate activity was displayed by extracts of biomass treated, for 72 and 120 h, with 2 g/L of this precursor (17.63 ± 0.08 mg TE/g extract and 18.82 ± 0.23 mg TE/g extract) (Table 10).

In Table 11, the chelating ability of extracts obtained from biomass elicited with MeJa, CaCl_2_, AgNO_3_, and YE extract is reported. The extract obtained from the biomass elicited with MeJa for 24 h at a concentration of 10 µM was found to be the best, as demonstrated by an IC_50_ value equal to 0.51 ± 0.01 mg/mL (Table 11).

The extracts of biomass elicited with CaCl_2_ showed no chelating activity at any of the concentrations and times tested (Table 11). Regarding the abiotic elicitor AgNO_3_, the extracts of biomass treated with this compound showed weak activity; the extracts obtained from shoot culture treated with 1 mM of AgNO_3_ at each time of exposition and with 2 mM after 48 h of incubation were found to be active. The extract of biomass elicited with yeast showed poor chelating activity, as demonstrated by IC_50_ > 2 mg/mL (Table 11). On the contrary, the extracts obtained from biomass grown on a medium supplemented with precursors (Phenylalanine and Tyrosine) showed strong chelating activity. In particular, with regard to Phe, the best result was observed for the extract of biomass fed with a concentration of 2 g/L of this precursor for 168 h, as highlighted by IC_50_ (0.85 ± 0.01 mg/mL). Regarding Tyr, the extracts obtained after the supplementation of this precursor in concentrations of 1 and 2 g/L for 72 and 120 h showed strong chelating activity. Nevertheless, the most effective extract was that obtained from biomass fed with Tyr (for 72 h) at a dose of 2 g/L, with values of IC_50_ equal to 0.27 ± 0.01 mg/mL (Table 12).

## 4. Discussion

*Isatis tinctoria*, a species belonging to the Brassicaceae family, has long been utilized as an ornamental crop and for animal feeding and also has a documented history as an indigo dye source and medicinal plant. It also has an increasing importance in the cosmetic industry. The main class of secondary metabolites detected in polar extracts of *I. tinctoria* leaves is represented by phenolic compounds: flavonoids and phenolic acids [31,37]. Identified flavonoids are derivatives of flavones and flavonols (i.e., vicenin-2, stellarin-2, isovitexin luteolin-glucuronide, quercetin, buddleoside, isoscoparin, and apigenin glucosides), as well as flavonoids esterified with p-coumaric, ferulic, or sinapic acid (4′-O-feruloyl iscoscoparin-3″-O-glucoside-7-O-glucoside, 2″-O-feruloyl iscoscoparin-3″-O-glucoside-7-O-glucoside, iscoscoparin-3″-O-glucoside-7-O-feruoylglucoside, isoscoparin-3′-O-feruoylglucoside, isoscoparin 3′-O-sinapoylglucoside, and isoscoparin-3′-O-p-coumaroyl glucoside) [31,37]. Among phenolic acids, sinapic and ferulic acids are dominant in leaf extracts, as are neochlorogenic, chlorogenic, caffeic, p-coumaric, ferulic, and coumarylquinic acids [6,8,31].

Phenolic compounds are among the most important antioxidant constituents [38]. They act as promoters of human health through their scavenging activity by preventing chronic diseases such as cardiovascular diseases, cancers, type 2 diabetes, and neurodegenerative diseases [39]. Therefore, it is necessary to find an effective technique to obtain plant extracts with high levels of the desired antioxidants. In this context, in vitro shoot cultures of *I. tinctoria* were used to produce antioxidant compounds. Hence, the in vitro shoot culture was established, starting from the seed of this species. The best growth conditions recorded were the optimum, which was represented by the MS variant, containing 2.0/1.0 mg/L of BAP/NAA (an 11.663-fold increase compared to the initial inoculum); good increases (about 9-fold) were also highlighted for shoot cultures grown on variants containing 1.0/1.0 and 1.0/0 mg/L of BAP/NAA. In the existing literature, only one study on the shoot cultures of *I. tinctoria* is available [18]. The shoot cultures of this species were established from the nodal segments of young plants cultured on Gamborg (B5) medium supplemented with 1 mg/L of BAP; whereas, the addition of NAA to the medium did not increase shoot multiplication [18]. Our results are quite different, demonstrating that supplementation using both plant growth regulators, BAP and NAA, at the same concentration gave better growth than the addition of BAP alone.

Regarding the TPC, the results were contrary to what happened with the growth of the biomass, as it was found to be lower in the extract obtained from the shoot culture grown on the MS variant (2.0/1.0 mg/L of BAP/NAA) when compared to the TPC observed in the extract from the biomass grown on the variant containing 1.0/1.0 mg/L of BAP/NAA. Among all the extracts, the latter showed the best chelating activity and good radical scavenging properties—superimposable to those recorded for the extract from the variant containing 1.0/0 mg/L of BAP/NAA. Based on these results, the biomass grown on the latter MS variant (BAP/NAA 1.0/1.0) was selected for the elicitation experiments and those with the use of precursors.

Elicitation is recognized as the most practically feasible strategy for increasing the production of desirable secondary metabolites from cell, organ, and plant systems [40,41,42]. This process occurs in response to stress stimuli that activate the protective mechanisms of the plants via secondary metabolite biosynthesis. Elicitors are signals or molecules that can stimulate stress responses in plants, leading to the enhanced synthesis and accumulation of secondary metabolites or the induction of novel ones [43]. The use of elicitors for increasing the accumulation of phenolics has been reported in many plant species [28,44,45,46,47].

In this study, the effect of different elicitors on the in vitro production of phenolic compounds from *I. tinctoria* shoot culture was evaluated; different concentrations and incubation times were considered to increase the yield of these compounds in the extracts prepared from the elicited biomass. The selection of the elicitation conditions should be carried out experimentally, with each time including a wide range of tested factors. In addition to the exposure time, type, and concentration of the elicitor, the type of culture and its sensitivity to environmental factors are extremely important.

HPLC analysis, performed to determine phenolic compounds in the elicited biomass, led to the identification of six flavonoids (vicenin-2, isovitexin, apigetrin, apigenin, quercitrin, quercetin) and nine phenolic acids (neochlorogenic, protocatechuic, chlorogenic, vanillic, caffeic, syringic, p-coumaric, ferulic, sinapic acids). These phenolic compounds were identified also in the hydroalcoholic extract obtained from the leaves of the parent plant, except for the glycoside flavonoid quercitrin and the phenolic acids vanillic and syringic acids, which were detected only in the elicited biomass [1]. These data are not surprising because in vitro cultures, especially if elicited or fed with precursors, very frequently synthesize compounds that are not produced in vivo. This depends on the species of the studied plant and on the specificity of cellular metabolism. In vitro cultures often carry out the glycosylation process more intensively; thus, an increased production of many glycosylated derivatives of metabolites is observed. In some cases, metabolism is directed to the production of a major species-specific metabolite, and the elicitation or administration of precursors enhances this biosynthesis.

From a quantitative point of view, the highest amount of phenolic compounds was found in the extracts of biomass grown on a medium supplemented with 1 g/L of Tyr for 120 and 168 h. These extracts were the richest in phenolic compounds with a superimposable total content of flavonoids (28.085 and 27.701 mg/g extract) and phenolic acids (10.279 and 11.592 mg/g extract). These data agree with the results of spectrophotometric determinations, resulting in the richest concentration of TPC (58.65 and 60.36 mg GAE/g extract).

Considering that Tyr supplementation (1 g/L, 168 h) caused a negative effect on growth, on the contrary, the same dose, after 120 h of incubation, determined a 1.54-fold increase in the biomass growth. The best result is represented by the supplementation with the lowest concentration of this precursor for an incubation time of 120 h. Notably, the total amount of the phenolic acids and flavonoids accumulated in in vitro biomass supplemented with Tyr (1 g/L, 120 h of incubation) was higher than that of the parent plant. Neochlorogenic, caffeic, ferulic, and sinapic acids were found to be increased by 5.82, 2.36, 1.57, and 1.80-fold. The flavonoids vicenin 2, isovitexin, and quercetin were increased by 1.44, 2.40, and 4.37-fold, in comparison with the cauline leaf extract of the parental plant [31]. This is direct evidence of the cellular uptake of Tyrosine from the liquid medium and its incorporation into the biosynthetic pathways of phenolic metabolites. Tyrosine, similar to Phenylalanine, is formed from chorismic acid and can be the precursor of a wide range of phenolic metabolites [27]. However, Tyrosine feeding is not a procedure that is often used in plant biotechnology. It is also less convenient because of its lower solubility in water and the pH of aqueous solutions. We previously tested Tyrosine as a precursor of flavonoids and phenolic compounds in agar and agitated cultures of *Scutelaria* sp. In the case of *S. lateriflora*, the use of Tyrosine in agar cultures causes a double decrease in the content of flavonoids but, in contrast, increases the accumulation of verbascoside. In agitated cultures, no positive effect for flavonoids was observed. Furthermore, Tyrosine administration had a negative effect on biomass growth and appearance [28]. A different result was obtained in cultures of *S. baicalensis,* in which Tyrosine administration resulted in a higher content of flavonoids (approximately 7 times more) and verbascoside (2 times more) compared to the control [48]. For this culture, as for *Isatis*, this was the best strategy to increase metabolite production.

HPLC analysis demonstrated that the best approach to enhance the accumulation of phenolic compounds in *I. tinctoria* shoot culture is in the form of supplementation with precursors, and in particular, with Tyrosine, which, at the lowest dose and after 120 or 168 h of exposure, facilitated a notable increase in phenolic compounds. Positive but lower effects, compared to the use of the precursors, were also observed for the CaCl_2_ elicitor (20 mM, 24 h), which yielded the best results among the elicitors, followed by MeJa (50, 100 µM, 120 h).

Recent studies have reported the in vitro production of flavonoids from *I. tinctoria* hairy root cultures elicited via different means, such as via chitosan, ultraviolet radiation (UV), the biotic elicitor *Aspergillus niger*, and *A. oryzae*, as well as salicylic acid and methyl jasmonate. In the elicitation experiment with chitosan, an increase in the total flavonoid content (16.35 mg/g root DW) was recorded [14]. The production of flavonoids in *I. tinctoria* hairy root cultures was also increased after exposure to UV-B radiation (7.259 mg/g root DW.) [15], as well as by treatment with *Aspergillus niger* and *A. oryzae* (3.018 mg/g root DW) [16]. A recent work on the elicitation of *I. tinctoria* hairy root cultures reported an enhanced production of flavonoids after abiotic elicitation with salicylic acid and methyl jasmonate [17].

The antioxidant properties of all the extracts of elicited biomass were evaluated by different in vitro methods; the primary antioxidant properties were examined using the DPPH assay, while the secondary antioxidant ability was determined by measuring the ferrous ion (Fe^2+^)-chelating activity.

The best radical scavenging was demonstrated by the extracts of biomass elicited with MeJa (50 and 100 µM, 120 h) and CaCl_2_ (50 mM, 24 h). These extracts contain a high quantity of vicenin, isovitexin, apigenin, and quercetin, as well as caffeic, syringic, and vanillic acids. It can be hypothesized that the strong antioxidant properties of the extracts could be related to these flavones but mainly could depend on the quercetin, which is a powerful radical scavenger [49,50,51,52]. The activity highlighted in the DPPH test might be also attributable to phenolic acids, especially hydroxycinnamic acids, in particular caffeic acid, which, presenting 2 hydroxyl groups, has strong radical scavenging properties [52].

With regards to the chelating activity, the extract with the best efficacy was seen in the biomass supplemented with Tyr 2 g/L for 72 h. Although this extract has a lower total flavonoid content than the extracts obtained from biomass supplemented with Tyr 1 g/L, it is the richest in chlorogenic acid (1156 mg/g extract), whose chelating properties have long been recognized [53]. Therefore, the strong chelating activity of this extract could be related to all of the contained phenolic compounds, but mainly to the chlorogenic acid [53]. The notion that other compounds contained in the extracts may also contribute to the observed effects cannot be disregarded.

All of these findings demonstrate that the addition of hormonal MeJa or abiotic elicitors, such as CaCl_2_, as well as the addition of the precursor Tyrosine, can boost the biosynthesis and accumulation of antioxidant compounds in in vitro cultures of *I. tinctoria*. On the other hand, the administration of Tyrosine to the medium for long periods (120 and 168 h) caused the darkening of the biomass, and had some negative effects on its growth. This disadvantage could be overcome by improving the nutrient and oxygen exchanges of the shoot culture by using bioreactors.

Other studies are underway to evaluate the effect of different types of bioreactors, including balloon, stirred-tank, and Plantform bioreactors, on biomass growth and the bioactive metabolites’ production in *Isatis tinctoria* shoot culture. Further studies are in progress to evaluate whether exposure to different types of light can further enhance the biosynthesis of antioxidant compounds in the in vitro cultures of this species.

## 5. Conclusions

The present study is the first to report the establishment of shoots in the in vitro culture of *Isatis tinctoria* for the efficient production of valuable phenolic compounds, i.e., flavonoids and phenolic acids. Under the optimal culture conditions (MS medium, BAP/NAA 1.0/1.0 mg/L) and by utilizing Tyrosine as a precursor and/or MeJa and CaCl_2_ as elicitors, the total amount of the phenolic acids and flavonoids accumulated in in vitro biomass was higher than that of the parent plant. Therefore, this study highlights the utilization of *I. tinctoria* in vitro shoot culture as a promising biological system to produce extracts rich in antioxidant compounds.

## Figures and Tables

**Table 1:** Effect of different MS medium variants supplemented with different concentrations of BAP/NAA (2.0/1.0, 1.0/0.5, 1.0/1.0, 1.0/0, 0.5/0, 0/1.0 mg/L) on *Isatis tinctoria* in vitro stationary culture growth. Values are expressed as the mean ± SD (*n* = 5).

GRs BAP + NAA (mg/L)	Fresh Weight (g) After 2 Weeks	Dried Weight (g) After 2 Weeks	Time Fold
2.0/1.0	3.371 ± 0.154 ^a^	0.258 ± 0.032 ^a^	3.189 ± 0.487 ^a^
1.0/0.5	3.467 ± 0.386 ^a^	1.011 ± 0.022 ^a^	3.295 ± 0.369 ^a^
1.0/1.0	3.797 ± 0.750 ^a^	0.290 ± 0.045 ^a^	3.579 ± 0.705 ^a^
0/1.0	1.959 ± 0.363 ^b^	0.173 ± 0.027 ^b^	1.840 ± 0.345 ^b^
1.0/0	2.785 ± 0.794 ^a,b^	0.187 ± 0.069 ^a,b^	2.622 ± 0.752 ^a,b^
0.5/0	3.538 ± 0.827 ^a^	0.264 ± 0.036 ^a^	3.321 ± 0.775 ^a^

Time fold (the final weight divided by inoculum weight) is referred to as fresh weight. Different letters within the same column indicate significant differences (*p* < 0.05) based on one-way ANOVA followed by Tukey–Kramer multiple comparisons test.

**Table 2:** Effect of different MS medium variants supplemented with different concentrations of BAP/NAA (2.0/1.0, 1.0/0.5, 1.0/1.0, 1.0/0, 0.5/0, 0/1.0 mg/L) on *Isatis tinctoria* in vitro agitated culture growth. Values are expressed as the mean ± SD (*n* = 6).

PGRs BAP + NAA (mg/L)	Fresh Weight (g) After 2 Weeks	Dried Weight (g) After 2 Weeks	Time Fold
2.0/1.0	5.927 ± 2.014 ^a^	0.447 ± 0.134 ^a^	11.663 ± 4.046 ^a^
1.0/0.5	4.424 ± 3.353 ^a,b^	0.425 ± 0.212 ^a^	8.654 ± 6.586 ^a,b^
1.0/1.0	5.038 ± 2.272 ^a^	0.401 ± 0.146 ^a^	9.973 ± 4.524 ^a^
0/1.0	0.996 ± 0.589 ^b^	0.092 ± 0.051 ^b^	1.964 ± 1.154 ^b^
1.0/0	5.589 ± 1.511 ^a^	0.467 ± 0.110 ^a^	11.027 ± 2.996 ^a^
0.5/0	3.092 ± 1.646 ^a,b^	0.223 ± 0.118 ^a,b^	6.117 ± 3.252 ^a,b^

Time fold (the final weight divided by inoculum weight) is referred to as fresh weight. Different letters within the same column indicate significant differences (*p* < 0.05) based on one-way ANOVA followed by Tukey–Kramer multiple comparisons test.

**Table 3:** Effect of different elicitors treatment on *Isatis tinctoria* in vitro agitated cultures growth (MS variant BAP/NAA 1.0/1.0 mg/L). Values are expressed as the mean ± SD (*n* = 3).

Elicitor		Dry Biomass Weight (g)			
		24 h	48 h	120 h	168 h
MeJa	10 µM	1.324 ± 0.097	0.586 ± 0.381	1.216 ± 0.447	0.678 ± 0.371
	50 µM	1.230 ± 0.025	1.076 ± 0.741	0.967 ± 0.754	0.335 ± 0.195
	100 µM	1.329 ± 0.239	0.961 ± 0.847	0.922 ± 0.600	0.463 ± 0.649
	Ctr EtOH 50%	0.830 ± 0.404	0.784 ± 0.345	1.064 ± 0.634	0.379 ± 0.058
CaCl_2_	20 mM	1.215 ± 0.552	0.251 ± 0.096	0.258 ± 0.116	0.305 ± 0.067
	50 mM	1.553 ± 0.060	0.273 ± 0.038	0.317 ± 0.093	0.556 ± 0.120
	100 mM	1.038 ± 0.832	0.346 ± 0.157	0.383 ± 0.252	0.537 ± 0.184
	Ctr H_2_O	0.746 ± 0.650	0.490 ± 0.354	0.836 ± 0.636	0.835 ± 0.616
AgNO_3_	0.5 mM	1.112 ± 0.166	0.684 ± 0.242	0.430 ± 0.057	
	1 mM	1.347 ± 0.144	1.306 ± 0.126	0.965 ± 0.276	
	2 mM	0.660 ± 0.351	0.709 ± 0.092	0.618 ± 0.185	
	Ctr H_2_O	0.746 ± 0.650	0.490 ± 0.354	0.836 ± 0.636	
YE extract	50 mg/L		0.609 ± 0.150	0.422 ± 0.171	0.515 ± 0.069
	200 mg/L		0.378 ± 0.028	0.532 ± 0.345	0.707 ± 0.201
	300 mg/L		0.410 ± 0.142	0.534 ± 0.267	0.567 ± 0.051
	Ctr H_2_O		0.490 ± 0.354	0.836 ± 0.636	0.835 ± 0.616

MeJa—methyl jasmonate; YE—yeast.

**Table 4:** Effect of precursors (L-Phenylalanine and L-Tyrosine) on *Isatis tinctoria* in vitro agitated cultures growth (MS variant BAP/NAA 1.0/1.0 mg/L). Values are expressed as the mean ± SD (*n* = 3).

Precursor (g/L)		Dry Biomass Weight (g)		
		72 h	120 h	168 h
Phe	1	1.210 ± 0.637	1.358 ± 0.246	0.852 ± 0.334
	2	1.290 ± 0.684	1.096 ± 0.203	1.136 ± 0.225
	Ctr H_2_O	0.426 ± 0.083	0.836 ± 0.636	0.835 ± 0.616
Tyr	1	1.485 ± 0.225	1.288 ± 0.183	0.643 ± 0.260
	2	1.082 ± 0.269	0.779 ± 0.319	0.319 ± 0.098
	Ctr H_2_O	0.426 ± 0.083	0.836 ± 0.636	0.835 ± 0.616

Phe—Phenylalanine; Tyr—Tyrosine.

**Table 5:** Determination of total phenolic content (calculated as gallic acid equivalents) of hydroalcoholic extracts obtained from *Isatis tinctoria* agitated shoot cultures grown on different MS variants. Values are expressed as the mean ± SD (*n* = 3).

MS Medium Variant BAP/NAA (mg/L)	Total Phenolic Content (TPC) mg GAE/g Extract (DW)
2.0/1.0	13.833 ± 0.506 ^a,b^
1.0/0.5	13.306 ± 0.973 ^a,b^
1.0/1.0	14.451 ± 1.043 ^a^
1.0/0	12.411 ± 0.566 ^b^
0.5/0	12.818 ± 0.161 ^b^
0/1.0	9.604 ± 0.555 ^c^

GAE—gallic acid equivalents. Different letters within the same column indicate significant differences (*p* < 0.05) based on one-way ANOVA followed by Tukey–Kramer multiple comparisons test.

**Table 6:** Determination of total phenolic content (calculated as gallic acid equivalent) of hydroalcoholic extracts obtained from *Isatis tinctoria* elicited shoot cultures (MS variant BAP/NAA 1.0/1.0 mg/L). Values are expressed as the mean ± SD (*n* = 3).

Elicitor		Total Phenolic Content (TPC) mg GAE/g Extract DW			
		24 h	48 h	120 h	168 h
MeJa	10 µM	18.91 ± 0.21 ^aA^	11.92 ± 0.20 ^aB^	24.96 ± 0.34 ^aC^	16.27 ± 0.07 ^aA^
	50 µM	22.58 ± 1.15 ^abA^	24.82 ± 0.69 ^bA^	33.36 ± 1.65 ^bB^	16.14 ± 0.27 ^aC^
	100 µM	22.99 ± 0.46 ^abA^	26.91 ± 2.09 ^bAB^	28.42 ± 2.95 ^aB^	17.45 ± 0.28 ^aC^
	CtrEtOH50%	16.80 ± 1.54 ^acA^	19.24 ± 1.01 ^cA^	36.54 ± 0.22 ^bB^	23.19 ± 0.55 ^bC^
CaCl_2_	20 mM	30.43 ± 1.06 ^aA^	20.52 ± 0.27 ^aB^	25.51 ± 0.02 ^aC^	29.51 ± 1.66 ^aD^
	50 mM	29.87 ± 1.07 ^aA^	26.51 ± 0.54 ^bB^	19.15 ± 1.32 ^bC^	31.57 ± 1.47 ^aA^
	100 mM	25.73 ± 0.26 ^bA^	16.67 ± 1.55 ^cB^	19.80 ± 0.65 ^bC^	24.13 ± 0.79 ^bA^
	Ctr H_2_O	24.74 ± 0.84 ^bA^	20.23 ± 0.86 ^aB^	29.56 ± 1.44 ^cC^	32.93 ± 0.42 ^aC^
AgNO_3_	0.5 mM	17.85 ± 0.72 ^aA^	17.04 ± 0.62 ^aA^	19.79 ± 0.07 ^aA^	
	1 mM	18.67 ± 0.86 ^aA^	14.88 ± 0.14 ^bB^	17.75 ± 0.51 ^bA^	
	2 mM	15.68 ± 0.26 ^bA^	13.52 ± 0.74 ^bB^	16.30 ± 0.42 ^bA^	
	Ctr H_2_O	24.74 ± 0.84 ^cA^	20.23 ± 0.86 ^dB^	29.56 ± 1.44 ^cC^	
YE extract	50 mg/L		18.85 ± 0.97 ^aA^	17.77 ± 0.03 ^aA^	20.70 ± 0.02 ^aA^
	200 mg/L		18.93 ± 3.12 ^aA^	17.50 ± 0.19 ^aA^	15.57 ± 0.23 ^bA^
	300 mg/L		18.58 ± 0.10 ^aA^	16.03 ± 0.18 ^bA^	20.91 ± 0.46 ^aA^
	Ctr H_2_O		20.23 ± 0.86 ^aA^	29.56 ± 1.44 ^cB^	32.93 ± 0.42 ^cC^

GAE—gallic acid equivalents; DW—dry weight; MeJa—methyl jasmonate; YE—yeast. Different lowercase letters indicate significant differences (*p* < 0.01) in the row between concentrations of each treatment; different uppercase letters indicate significant differences (*p* < 0.01) in columns between different incubation times, based on two-way ANOVA followed by Tukey–Kramer multiple comparisons test.

**Table 7:** Determination of total phenolic content (calculated as gallic acid equivalents) of hydroalcoholic extracts obtained from *Isatis tinctoria* precursors feeding shoot cultures (MS variant BAP/NAA 1.0/1.0 mg/L supplemented with precursor Phenylalanine or Tyrosine). Values are expressed as the mean ± SD (*n* = 3).

Precursor (g/L)		Total Phenolic Content (TPC) mg GAE/g Extract DW		
		72 h	120 h	168 h
Phe	1	27.28 ± 1.20 ^aA^	36.00 ± 0.11 ^aB^	25.73 ± 1.14 ^aA^
	2	36.36 ± 2.76 ^bA^	32.585 ± 1.00 ^aA^	41.43 ± 6.45 ^bB^
	Ctr H_2_O	17.75 ± 0.48 ^cA^	29.556 ± 1.44 ^aB^	32.93 ± 0.42 ^abB^
Tyr	1	44.48 ± 1.15 ^aA^	58.65 ± 0.91 ^aB^	60.36 ± 4.97 ^aB^
	2	49.37 ± 0.93 ^aA^	36.52 ± 6.99 ^bB^	34.97 ± 0.45 ^bB^
	Ctr H_2_O	17.75 ± 0.48 ^bA^	29.56 ± 1.44 ^cB^	32.93 ± 0.42 ^bB^

Phe—Phenylalanine; Tyr—Tyrosine. Different lowercase letters indicate significant differences (*p* < 0.01) in the row between concentrations of each treatment; different uppercase letters indicate significant differences (*p* < 0.01) in columns between different incubation times, based on two-way ANOVA followed by Tukey–Kramer multiple comparisons test.

**Table 8:** Free radical scavenging activity (DPPH test), ferrous ion (Fe^2+^)-chelating activity of hydroalcoholic extracts obtained from *Isatis tinctoria* agitated shoot cultures grown on different MS variants. Values are expressed as the mean ± SD (*n* = 3).

MS Medium Variant BAP/NAA (mg/L)	DPPH Test mg TE/g Extract	Chelating Activity IC_50_ (mg/mL)
2.0/1.0	7.38 ± 0.34 ^a,c^	0.80 ± 0.05 ^a,e^
1.0/0.5	7.98 ± 0.45 ^a^	0.83 ± 0.07 ^a^
1.0/1.0	9.65 ± 0.26 ^b^	0.64 ± 0.01 ^b^
1.0/0	9.81 ± 0.69 ^b^	1.01 ± 0.01 ^c^
0.5/0	6.53 ± 0.33 ^c^	1.40 ± 0.06 ^d^
0/1.0	6.39 ± 0.42 ^c^	0.75 ± 0.01 ^e^

TE—Trolox equivalents. Different letters within the same column indicate significant differences (*p* < 0.05) based on one-way ANOVA followed by Tukey–Kramer multiple comparisons test.

**Table 9:** Determination of free radical scavenging activity (DPPH test) of hydroalcoholic extracts obtained from *Isatis tinctoria* elicited shoot cultures (MS variant BAP/NAA 1.0/1.0 mg/L). Values are expressed as the mean ± SD (*n* = 3).

Elicitor		DPPH Test mg TE/g Extract			
		24 h	48 h	120 h	168 h
MeJa	10 µM	14.90 ± 0.84 ^aA^	8.37 ± 0.70 ^aB^	18.90 ± 0.51 ^aC^	16.39 ± 0.64 ^aD^
	50 µM	15.48 ± 1.17 ^bA^	21.12 ± 0.78 ^bB^	24.20 ± 0.66 ^bC^	13.46 ± 0.09 ^bD^
	100 µM	17.11 ± 0.54 ^bA^	18.57 ± 0.37 ^cA^	24.79 ± 0.38 ^bB^	6.50 ± 0.03 ^cC^
	Ctr EtOH 50%	15.43 ± 0.51 ^bA^	14.28 ± 0.28 ^dA-^	25.27 ± 0.43 ^bB^	12.15 ± 0.59 ^bdC^
CaCl_2_	20 mM	21.91 ± 0.65 ^aA^	13.16 ± 0.34 ^aB^	16.81 ± 0.46 ^aC^	20.99 ± 0.91 ^aA^
	50 mM	25.14 ± 0.35 ^bA^	15.89 ± 0.49 ^bB^	11.35 ± 0.41 ^bC^	23.86 ± 0.84 ^bA^
	100 mM	19.22 ± 0.31 ^cA^	16.03 ± 0.51 ^bB^	12.38 ± 0.25 ^cC^	18.71 ± 1.07 ^cA^
	Ctr H_2_O	18.44 ± 0.01 ^cA^	18.01 ± 0.13 ^cA^	24.49 ± 0.088 ^dB^	25.24 ± 0.63 ^bC^
AgNO_3_	0.5 mM	11.08 ± 0.97 ^aA^	9.72 ± 0.78 ^aB^	6.62 ± 0.03 ^aC^	
	1 mM	9.20 ± 0.15 ^bA^	11.88 ± 0.19 ^bB^	10.92 ± 0.28 ^bB^	
	2 mM	7.12 ± 0.70 ^cA^	5.19 ± 0.17 ^cB^	4.25 ± 0.58 ^cB^	
	Ctr H_2_O	18.44 ± 0.01 ^dA^	18.01 ± 0.13 ^dA^	24.49 ± 0.09 ^dB^	
YE extract	50 mg/L		17.65 ± 0.46 ^aA^	19.73 ± 0.26 ^aB^	19.70 ± 0.44 ^aB^
	200 mg/L		12.28 ± 0.01 ^bA^	22.03 ± 0.79 ^bB^	15.54 ± 0.11 ^bC^
	300 mg/L		15.71 ± 0.31 ^cA^	19.01 ± 0.23 ^bB^	18.25 ± 0.01 ^cB^
	Ctr H_2_O		18.01 ± 0.13 ^aA^	24.49 ± 0.09 ^cB^	25.24 ± 0.63 ^dC^

MeJa—methyl jasmonate; TE—trolox equivalents; YE—yeast. Different lowercase letters indicate significant differences (*p* < 0.01) in the row between concentrations of each treatment; different uppercase letters indicate significant differences (*p* < 0.01) in columns between different incubation times, based on two-way ANOVA followed by Tukey–Kramer multiple comparisons test.

**Table 10:** Determination of free radical scavenging activity (DPPH test) of hydroalcoholic extracts obtained from *Isatis tinctoria* elicited shoot cultures (MS variant BAP/NAA 1.0/1.0 mg/L supplemented with precursor Phe or Tyr). Values are expressed as the mean ± SD (*n* = 3).

Precursor (g/L)		DPPH Test mg TE/g Extract		
		72 h	120 h	168 h
Phe	1	12.70 ± 0.02 ^aA^	19.36 ± 0.94 ^aB^	12.4 ± 0.45 ^aA^
	2	19.51 ± 0.55 ^bA^	21.85 ± 0.15 ^bB^	17.44 ± 0.03 ^bC^
	Ctr H_2_O	7.23 ± 0.40 ^cA^	24.49 ± 0.09 ^cB^	25.24 ± 0.63 ^cB^
Tyr	1	13.98 ± 0.90 ^aA^	16.27 ± 0.35 ^aB^	17.49 ± 0.04 ^aB^
	2	17.63 ± 0.08 ^bA^	18.82 ± 0.23 ^bA^	6.70 ± 0.95 ^bB^
	Ctr H_2_O	7.23 ± 0.40 ^cA^	24.49 ± 0.09 ^cB^	25.24 ± 0.63 ^cB^

Phe—Phenylalanine; Tyr—Tyrosine; TE—trolox equivalents. Different lowercase letters indicate significant differences (*p* < 0.01) in the row between concentrations of each treatment; different uppercase letters indicate significant differences (*p* < 0.01) in columns between different incubation times, based on two-way ANOVA followed by Tukey–Kramer multiple comparisons test.

**Table 11:** Determination of ferrous ion (Fe^2+^)-chelating activity of hydroalcoholic extracts obtained from *Isatis tinctoria* elicited shoot cultures (MS variant BAP/NAA 1.0/1.0 mg/L). Values are expressed as the mean ± SD (*n* = 3).

Elicitor		Chelating Activity IC_50_ (mg/mL)			
		24 h	48 h	120 h	168 h
MeJa	10 µM	0.51 ± 0.01 ^aA^	1.57 ± 0.04 ^aB^	0.64 ± 0.02 ^aC^	>2 ^aD^
	50 µM	0.94 ± 0.01 ^bA^	1.32 ± 0.01 ^bB^	1.22 ± 0.02 ^bC^	>2 ^aD^
	100 µM	1.17 ± 0.01 ^cA^	1.11 ± 0.02 ^cA^	0.99 ± 0.02 ^cB^	Na
	Ctr EtOH 50%	0.89 ± 0.01 ^bA^	0.91 ± 0.01 ^dA^	0.76 ± 0.01 ^dB^	1.18 ± 0.02 ^bC^
CaCl_2_	20 mM	Na	Na	Na	Na
	50 mM	Na	Na	Na	Na
	100 mM	Na	Na	Na	Na
	Ctr H_2_O	>2 ^A^	>2 ^A^	0.97 ± 0.01 ^B^	1.74 ± 0.04 ^C^
AgNO_3_	0.5 mM	>2 ^a^	>2 ^a^	>2 ^a^	
	1 mM	1.53 ± 0.02 ^bA^	0.76 ± 0.05 ^bB^	1.35 ± 0.02 ^bC^	
	2 mM	>2 ^aA^	1.14 ± 0.03 ^cB^	>2 ^aA^	
	Ctr H_2_O	>2 ^cA^	>2 ^aA^	0.97 ± 0.01 ^cB^	
YE extract	50 mg/L		>2 ^a^	>2 ^a^	>2 ^a^
	200 mg/L		>2 ^a^	>2 ^a^	>2 ^a^
	300 mg/L		Na	Na	Na
	Ctr H_2_O		>2 ^aA^	0.97 ± 0.01 ^bB^	1.74 ± 0.04 ^bC^

MeJa—methyl jasmonate; Na—not active. Different lowercase letters indicate significant differences (*p* < 0.01) in the row between concentrations of each treatment; different uppercase letters indicate significant differences (*p* < 0.01) in columns between different incubation times, based on two-way ANOVA followed by Tukey–Kramer multiple comparisons test.

**Table 12:** Determination of ferrous ion (Fe^2+^)-chelating activity of extracts obtained from *Isatis tinctoria* elicited shoot cultures (MS variant BAP/NAA 1.0/1.0 mg/L supplemented with precursor Phe or Tyr).

Precursor (g/L)		Chelating Activity IC_50_ (mg/mL)		
		72 h	120 h	168 h
Phe	1	1.48 ± 0.05 ^aA^	1.33 ± 0.03 ^aB^	1.63 ± 0.05 ^aC^
	2	1.06 ± 0.01 ^bA^	1.05 ± 0.03 ^bA^	0.85 ± 0.01 ^bB^
	Ctr H_2_O	>2 ^cA^	0.97 ± 0.01 ^bB^	1.74 ± 0.04 ^cC^
Tyr	1	0.54 ± 0.03 ^aA^	0.69 ± 0.02 ^aB^	1.42 ± 0.02 ^aC^
	2	0.27 ± 0.01 ^bA^	0.57 ± 0.03 ^bB^	1.86 ±0.07 ^bC^
	Ctr H_2_O	>2 ^cA^	0.97 ± 0.01 ^cB^	1.74 ± 0.04 ^cC^

Phe—Phenylalanine; Tyr—Tyrosine. Different lowercase letters indicate significant differences (*p* < 0.01) in the row between concentrations of each treatment; different uppercase letters indicate significant differences (*p* < 0.01) in columns between different incubation times, based on two-way ANOVA followed by Tukey–Kramer multiple comparisons test.

## Data Availability

Not applicable.

## Supplementary Materials

The following are available online at https://www.mdpi.com/article/10.3390/antiox12051111/s1, Table S1: Percentage yields of hydroalcoholic extracts obtained from *Isatis tinctoria* shoot cultures grown on different MS medium variants. Table S2: Percentage yields of hydroalcoholic extracts obtained from *Isatis tinctoria* elicited shoot cultures (MS variant BAP/NAA 1.0/1.0 mg/L); Table S3: Percentage yields of hydroalcoholic extracts obtained from *Isatis tinctoria* precursors feeding shoot cultures (MS variant BAP/NAA 1.0/1.0 mg/L supplemented with precursor Phe and Tyr). Figure S1: Content of phenolic acids and flavonoids, determined by RP-HPLC analysis, in *Isatis tinctoria* in vitro culture extracts elicited by Silver nitrate-AgNO_3_ (A,C) and yeast extract-YE (B,D). Values are expressed as the mean ± SD (*n* = 3).

## Abbreviations

ASE	Ascorbic acid equivalent
BAP	6-Benzylaminopurine
DPPH	2,2-diphenyl-1-picrylhydrazyl
DW	Dry Weight
GAE	Gallic Acid Equivalents
IBA	Indole-3-butyric acid
KIN	Kinetin
Tyr	L-Tyrosine
Phe	L-Phenylalanine
MeJa	Methyl Jasmonate
MS	Murashige and Skoog
NAA	1-naphthaleneacetic acid
TPC	Total Phenolic Content
TE	Trolox equivalents
YE	Yeast
