# Supplementary material for: Improvement in the Biosynthesis of Antioxidant-Active Metabolites in In Vitro Cultures of Isatis tinctoria (Brassicaceae) by Biotechnological Methods/Elicitation and Precursor Feeding
Source: Antioxidants (Basel). 2023 May 17;12(5):1111. doi: 10.3390/antiox12051111 (PMC10215496; doi:10.3390/antiox12051111)
Supplement: Supplementary file 1 [file antioxidants-12-01111-s001.zip › antioxidants-2367314-supplementary.pdf]

Improvement in the Biosynthesis of Antioxidant-Active Metabolites in In Vitro Cultures of *Isatis tinctoria* (Brassicaceae) by Biotechnological Methods/Elicitation and Precursor Feeding

Natalizia Miceli [1,*,†], Inga Kwiecień [2,*,†], Noemi Nicosia [3,4], Jasmine Speranza [3,5], Salvatore Ragusa [6], Emilia Cavò [1,3], Federica Davì [1,3], Maria Fernanda Taviano [1] and Halina Ekiert [2]

1 Department of Chemical, Biological, Pharmaceutical and Environmental Sciences, University of Messina, Viale Ferdinando Stagno d'Alcontres 31, 98166 Messina, Italy; emilia.cavo@unime.it (E.C.); federica.davi@unime.it (F.D.); mtaviano@unime.it (M.F.T.)
2 Department of Pharmaceutical Botany, Faculty of Pharmacy, Jagiellonian University Medical College, Medyczna 9 Str., 30-688 Krakow, Poland; halina.ekiert@uj.edu.pl
3 Foundation "Prof. Antonio Imbesi", University of Messina, Piazza Pugliatti 1, 98122 Messina, Italy;
nicosia.noemi@hsr.it (N.N.); js1101@leicester.ac.uk (J.S.)
4 Division of Neuroscience, Vita Salute San Raffaele University, 20132 Milan, Italy
5 Department of Genetics and Genome Biology, University of Leicester, University Road, Leicester LE1 7RH, UK
6 PLANTA/Research, Documentation and Training Center, Via Serraglio Vecchio 28, 90123 Palermo, Italy; sragusa@unicz.it
* Correspondence: nmiceli@unime.it (N.M.); inga.kwiecien@uj.edu.pl (I.K.)
† These authors contributed equally to this work.

Supplementary Materials

Table S1. Percentage yields of hydroalcoholic extracts obtained from *Isatis tinctoria* shoot cultures grown on different MS medium variants

MS Medium Variant BAP/NAA (mg/L)	Yield of extracts from *Isatis tinctoria* agitated shoot cultures (%)
2.0/1.0	43.17
1.0/0.5	36.73
1.0/1.0	51.40
1.0/0	49.17
0.5/0	48.71
0/1.0	61.20

Table S2. Percentage yields of hydroalcoholic extracts obtained from *Isatis tinctoria* elicited shoot cultures (MS variant BAP/NAA 1.0/1.0 mg/L).

Elicitors		Yield of extracts from *Isatis tinctoria* elicited shoot cultures (%)			
		24 h	48 h	120 h	168 h
MeJa	10 μM	44.38	36.76	33.85	28.66
	50 μM	40.56	33.35	23.23	19.90
	100 μM	41.58	37.28	31.19	11.96
	Ctr EtOH 50%	43.04	33.25	29.46	10.25
$CaCl_2$	20 mM	38.37	11.38	16.69	35.82
	50 mM	38.63	17.09	22.73	26.32
	100 mM	42.83	19.05	22.44	30.81
	Ctr H_2O	34.28	31.38	30.40	33.23
$AgNO_3$	0.5 mM	34.25	18	19.97	
	1 mM	34.21	27.5	20.33	
	2 mM	34.33	22	7	
	Ctr H_2O	34.28	31.38	30.40	
YE extract	50 mg/L		30.12	10.18	15.54
	200 mg/L		29.20	29.21	33
	300 mg/L		21.93	40.68	14.12
	Ctr H_2O		31.38	30.40	33.23

MeJa – methyl jasmonate; YE – Yeast extract

Table S3. Percentage yields of hydroalcoholic extracts obtained from *Isatis tinctoria* precursors feeding shoot cultures (MS variant BAP/NAA 1.0/1.0 mg/L supplemented with precursor Phe and Tyr).

Precursor (g/L)		Yield of extracts from *Isatis tinctoria* elicited shoot cultures (%)		
		72 h	120 h	168 h
Phe	1	26.17	40.90	23.90
	2	30.76	38.03	30.80
	Ctr H_2O	14.30	30.40	33.23
Tyr	1	50.71	35.06	12.27
	2	26.96	17.54	14
	Ctr H_2O	14.30	30.40	33.23

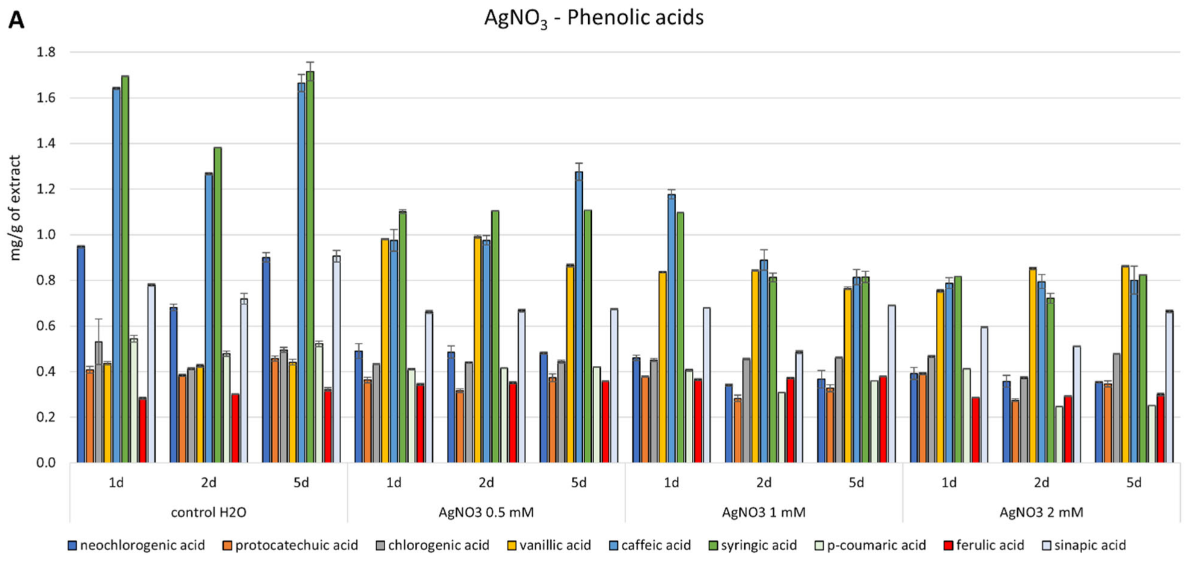
A
AgNO$_3$ - Phenolic acids
mg/g of extract
1.8
1.6
1.4
1.2
1.0
0.8
0.6
0.4
0.2
0.0
1d
2d
5d
control H2O
AgNO3 0.5 mM
AgNO3 1 mM
AgNO3 2 mM
neochlorogenic acid
protocatechuic acid
chlorogenic acid
vanillic acid
caffeic acid
syringic acid
p-coumaric acid
ferulic acid
sinapic acid

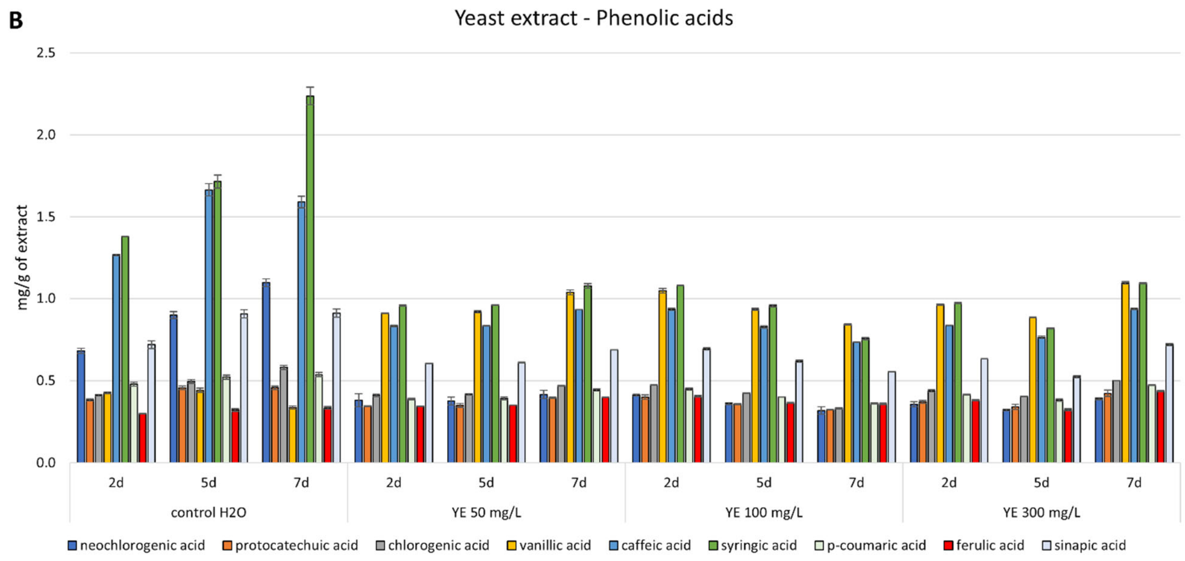
B
Yeast extract - Phenolic acids
mg/g of extract
2.5
2.0
1.5
1.0
0.5
0.0
2d
5d
7d
control H2O
YE 50 mg/L
YE 100 mg/L
YE 300 mg/L
neochlorogenic acid
protocatechuic acid
chlorogenic acid
vanillic acid
caffeic acid
syringic acid
p-coumaric acid
ferulic acid
sinapic acid

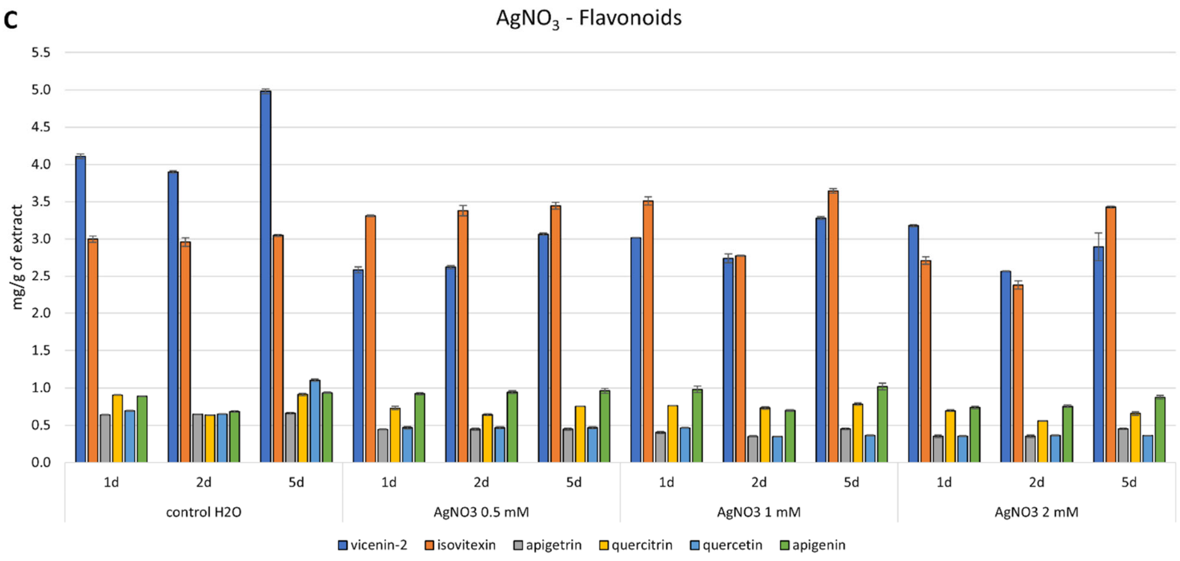

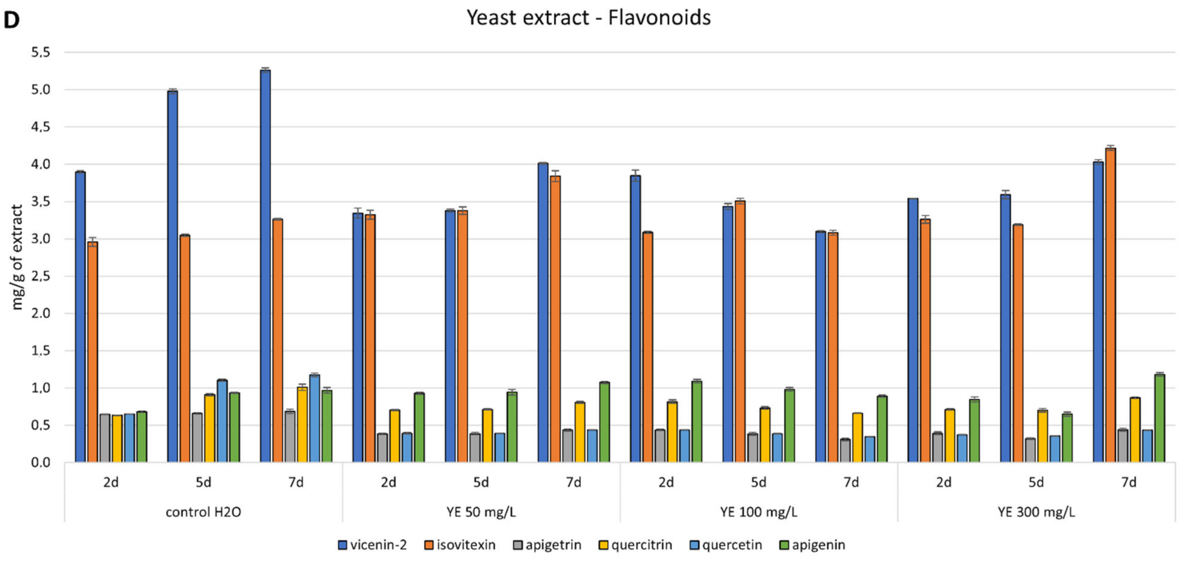

Figure S1. Content of phenolic acids and flavonoids, determined by RP-HPLC analysis, in *Isatis tinctoria in vitro* culture extracts elicited by Silver nitrate - $AgNO_3$(A, C), and Yeast extract – YE (B, D). Values are expressed as the mean ± SD (n = 3).
